# Reversible lung fibrosis in a 6-year-old girl after long term nitrofurantoin treatment

**DOI:** 10.1186/s12890-020-01353-x

**Published:** 2020-11-26

**Authors:** Lise Fischer Mikkelsen, Sune Rubak

**Affiliations:** Department of Paediatrics and Adolescent Medicine, Danish Center of Paediatric Pulmonology and Allergology, Aarhus University Hospital, Aarhus N, Denmark

**Keywords:** Nitrofurantoin, Side effects, Urinary tract infection, Lung fibrosis, Paediatrics

## Abstract

**Background:**

Pulmonary side effects are well known, including lung fibrosis, in elderly patients treated with long-term nitrofurantoin to prevent urinary tract infections and secondary renal injury. However, pulmonary side effects have only been reported rarely in paediatric cases, despite nitrofurantoin being a first line prophylactic treatment of recurrent childhood urinary tract infection.

**Case presentations:**

A 6-year-old girl was admitted to the hospital with dyspnea, general fatigue, loss of appetite and need for nasal oxygen treatment after long-term nitrofurantoin treatment. A computed tomography scan of the chest showed lung fibrosis. A biopsy confirmed this diagnosis. We suspected the fibrosis to be caused by the nitrofurantoin treatment. Thorough examinations reveal no other explanations. Nitrofurantoin was discontinued and the girl was treated with methylprednisolone. After 17 month a new scan and lung function test showed total regression of the lung fibrosis.

**Conclusions:**

This case underlines that risk of severe side effects should be taken in to account before initiation of long-term nitrofurantoin treatment in children.

## Background

Nitrofurantoin has been a first line antibiotic choice in prophylactic treatment of childhood urinary tract infection. Pulmonary toxicity causing irreversible pulmonary fibrosis is a well-known side effect of long-term nitrofurantoin treatment and other biological treatments in adults and elderly [1-4]. However, only few cases have been reported of pulmonary affection in children after nitrofurantoin treatment [5, 6].

We report a case of a 6-year-old girl who developed dyspnea and interim need for nasal oxygen treatment due to pulmonary fibrosis manifesting after 2 years of nitrofurantoin treatment.

## Case presentation

The 6-year-old girl was admitted to the hospital with dyspnea, general fatigue and loss of appetite developing during approximately 1 month. At admission, oxygen desaturation was 80–90% before nasal oxygen treatment.

Daily and continuous treatment with oral nitrofurantoin (tablets, 25 mg/day) in combination with solifenacin (tablets) had been initiated 2 years prior to the admission to prevent recurrent urinary tract infections. She presented no other medical history.

**Fig. 1:**
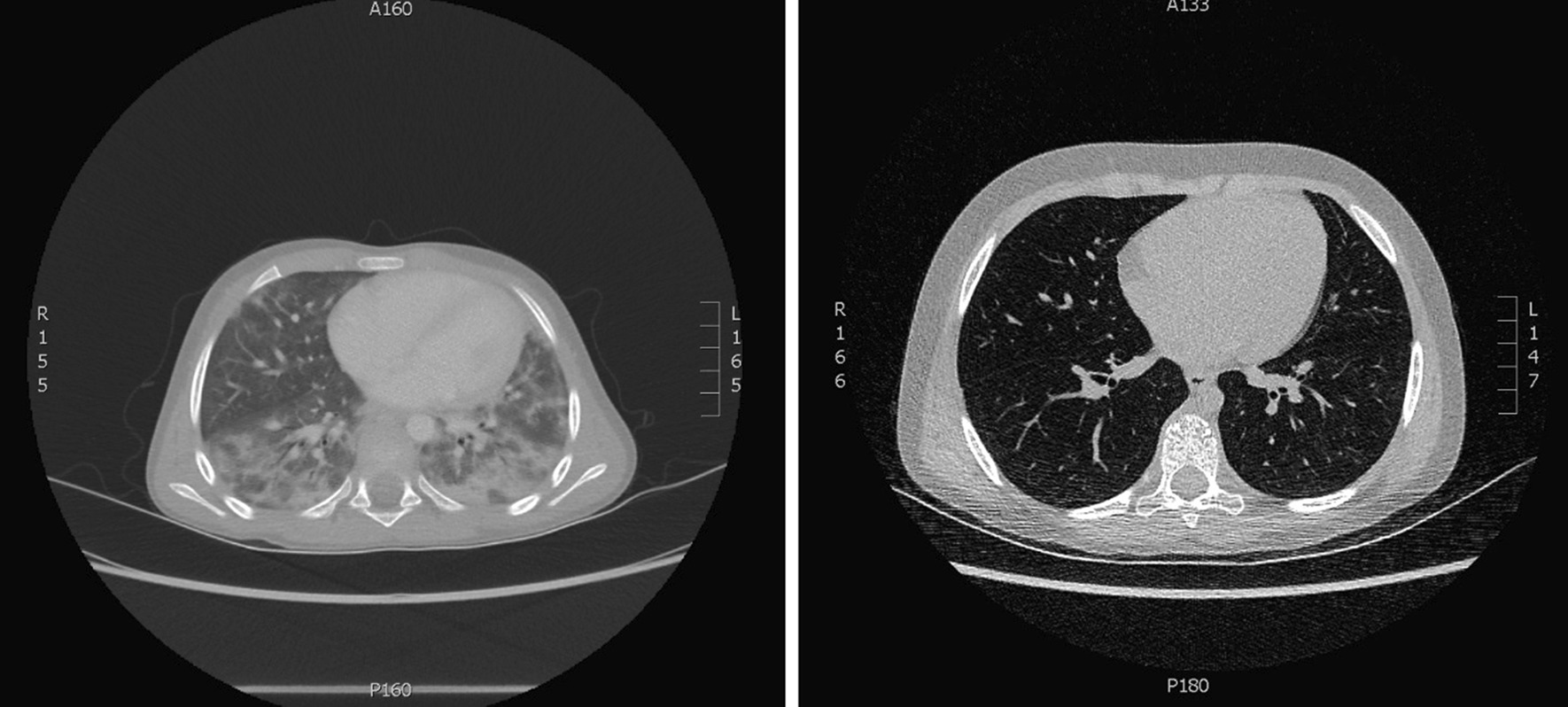
A computed tomography scan of the chest for the time of admission (left) showing bilateral multilobar parenchymal infiltrates, ground glass opacity, interstitial changes, and enlarged hilar lymph nodes however only discrete signs of lung fibrosis with no honeycomb change. After 8 months post high-dose methylprednisolone treatment (right) CT showing complete regression of lung findings

Initial blood samples revealed liver affection (p-lactic acid dehydrogenase was 199U/L and p-alanintransaminase was 750U/L), but otherwise biochemical parameters were unaffected. Multiple PCR analyses detected no microorganisms in samples from the upper respiratory tract. A computed tomography scan of the chest showed bilateral multilobar parenchymal infiltrates, ground glass opacity, interstitial changes, and enlarged hilar lymph nodes however only discrete signs of lung fibrosis with no honeycomb change, subpleural cysts or traction bronchiectasis (Fig. 1). Lung biopsy confirmed suspicion of drug induced lung fibrosis with chronic interstitial inflammation and microscopically diffuse alveolar damage and atypical distribution of the fibrosis involving both the lower and upper lobes bilateral.

On the suspected diagnosis of nitrofurantoin-induced pulmonary fibrosis and due to clinical deterioration including oxygen desaturation, dyspnea, restrictive pattern of lung function, initial treatment with intravenous methylprednisolone (35 mg/kg) was started at the day of admission. Nitrofurantoin and solifenacin were discontinued. After 3 days, treatment was changed to prednisolone tablets (20 mg/kg twice a day).

A bronchoscopy performed after 2 months of treatment due to ongoing respiratory symptomes and revealed structurally normal airways. However, microbiological analyses showed pneumocystis jirovecii in a broncho-alveolar lavage sample taken during the procedure. The infection was successfully treated with tablets of sulfametoxazol and trimetoprim (400 mg + 80 mg) three times a day for 3 weeks. It is difficult to be certain about the onset of pneumocystis jirovecii lung infection; however, this infection is most likely a consequence of immunosuppression due to steroid treatment or less likely secondary to the chronic lung changes.

After 2 months, prednisolone was withdrawn over a period of 25 days. However, a following synacthen test showed tertiary adrenal insufficiency and hydrocortisone replacement therapy was initiated.

**Fig. 2:**
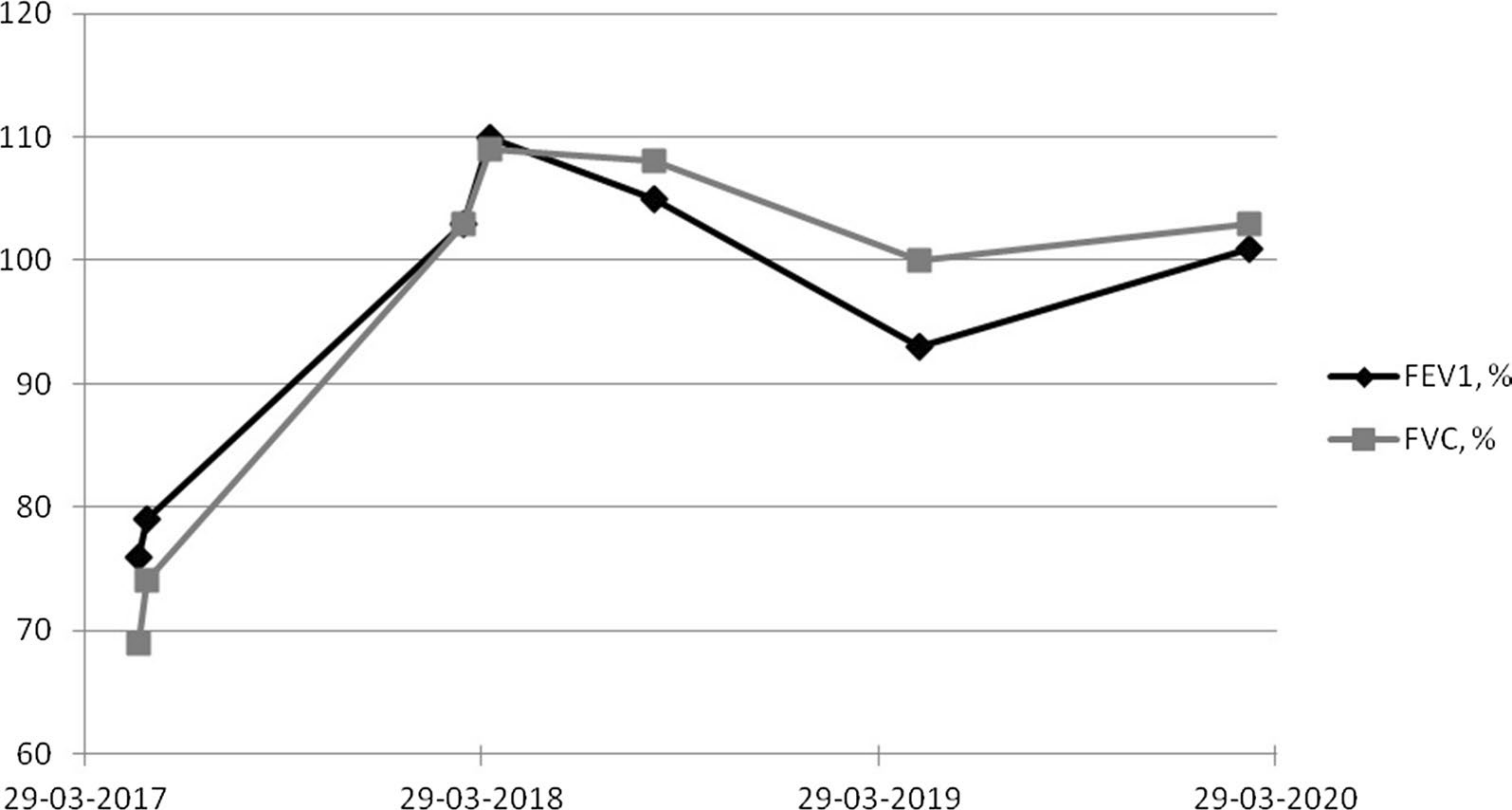
Progression of lung function over time, FVC and FEV1 in percent of predicted value. Diagnosis of lung fibrosis and discontinuation of nitrofurantoin and initiation of treatment with glucocorticoids at 29/3 2017

The girl was followed with frequent consultations and pulmonary function tests. Initial tests showed a restrictive pattern with reduced forced vital capacity (FVC) (69% of predicted value) and forced expiratory volume in 1 s (FEV1) (76% of predicted value), but no bronchial obstruction (FEV_1_/FCV-ratio unaffected). Following tests showed normalization of all parameters (FVC was 103% and FEV1 was 101% of predicted value) (Fig. 2). 17 months after the first admission, the girl performed a spirometry test showing normal pulmonary function and a high-rate computed tomography scan showed total regression of the pulmonary fibrosis.

## Discussion and conclusions

Nitrofurantoin is an antibiotic medicament often used in the treatment of recurrent urinary tract infections as the urinary excretion rate is high. Recent studies have questioned the efficacy of antibiotics in the prevention of recurrent urinary tract infections and secondary renal injury in children [7]. In a recent paper in the Lancet, the authors concluded that “a trial using antimicrobial prophylaxis in children with several recurrent episodes of acute pyelonephritis is warranted” [8].

In Denmark, the national paediatric society recently changed the national clinical guideline: Prophylactic antibiotics should only be prescribed by specialists and after treatment of relevant risk factors [9]. The recommendation is trimethoprim (2 mg/kg once a day) as first line treatment of recurrent upper urinary tract infection in children (amoxicillin if the child is younger than 6 weeks). Second choice is nitrofurantoin, demanding regular anamnestic screening of pulmonary symptoms including lung function testing.

The American Academy of Pediatrics (clinical guidelines, latest reaffirmation in 2016) recommends “prompt” initiation of antibiotic treatment in case of symptoms rather than prophylactic antibiotic [10] of recurrent urinary tract infection. Furthermore, the committee emphasizes the importance of treating bowl/bladder dysfunctions, as this is a major, but disregarded, risk factor for recurrent urinary tract infections.

Different pathophysiological mechanisms have been suggested to cause the pulmonary toxitcity of long-term nitrofurantoin treatment. One leading theory is that oxidative stress by the production of free radicals might injure the lung tissue, as nitrofurantoin in its active form is highly reactive. In vivo studies showed reduced injury in tissue incubated with nitrofurantoin in combination with antioxidants compared to nitrofurantoin alone. Hypersensitivity to nitrofurantoin and thereby cytokine-initiated inflammation is another possible explanation. However, hypersensitivity is more likely to cause acute reactions after short-time nitrofurantoin treatment [11].

In this case, a 6-year-old girl developed pulmonary fibrosis after 2 years of nitrofurantoin treatment preventing recurrent urinary tract infections. Thorough clinical examinations and paraclinical testing revealed no infection or other causes of the fibrosis at the time of admission. Discontinuation of nitrofurantoin and treatment of high-dose steroids resulted in full regression of the fibrosis and normalization of pulmonary symptoms and lung function. We conclude that the most probable cause to reversible lung fibrosis was the pulmonary toxicity of treatment with long-term nitrofurantoin. Whether or not the pneumocystis jirovecii infection verified in the lungs 2 months later had been ongoing for longer time is unknown, however if present at baseline and during steroid treatment it would have been expected to be associated with worsening of lung function.

This case shows that the well-known side effect of long-term nitrofurantoin treatment in elderly also may be a rare paediatric risk factor. Clinicians should consider alternative options when planning prophylactic treatment of recurrent urinary tract infections in children.

## Data Availability

Not applicable.

## Abbreviations

FVC: Forced vital capacity
FEV1: Forced expiratory volume in 1 s
